# Prevalence of hepatitis B virus in patients with diabetes mellitus: a comparative cross sectional study at Woldiya General Hospital, Ethiopia

**DOI:** 10.11604/pamj.2014.17.40.2465

**Published:** 2014-01-21

**Authors:** Daniel Mekonnen, Solomon Gebre-Selassie, Surafel Fantaw, Andualem Hunegnaw, Adane Mihret

**Affiliations:** 1 Bahir Dar Regional Health Research Laboratory Center, Bahir Dar, Ethiopia; 2 Department of Medical Microbiology, Immunology and Parasitology College of Health Science, School of Medicine, Addis Ababa University, Addis Ababa, Ethiopia; 3 Ethiopian Health and Nutrition Research Institute Bacteriology and Mycology Laboratory, Addis Ababa, Ethiopia; 4 Amhara National Regional State Health Bureau, Woldiya General Hospital, Woldiya, Ethiopia; 5 Armauer Hansen Research Institute, Addis Ababa, Ethiopia

**Keywords:** Prevalence, HBV, diabetes, non diabetes, association

## Abstract

**Introduction:**

The overall prevalence of HBV in Ethiopia varies from 4.7-16.8% for Hepatitis B surface antigen (HBsAg) and 70-76.38% for at least one marker positive. Patients suffering from type I Diabetes Mellitus (DM) incur high risk of infection with hepatotropic viruses because of frequent hospitalization and blood tests.

**Methods:**

A comparative cross sectional study was conducted at Woldiya General Hospital using 108 consented study populations from Diabetes and 108 non diabetes control groups during the period November 2010 - January 2011. VISITECT HBsAg rapid test kit and Humastat 80 chemistry analyzer were used. Multivariate logistic regression was used to see the association of HBV with clinical history of participants and Sociodemographic variables. All tests were two-sided with α-level of 0.05 and 80% power.

**Results:**

Prevalence of HBsAg was equal between diabetic and non diabetic individuals, 3.7% indicating that there was no difference between the two groups. Only history of invasive procedures and chronic liver disease showed association with HBsAg seropositivity.

**Conclusion:**

In this study a positive relation was not indicated between HBV and Diabetes and the prevalence of HBsAg was equal between diabetic and non diabetic individuals.

## Introduction

Hepatitis B Virus (HBV) infection and its sequelae (cirrhosis and liver cancer) are major global health problems. It has been estimated that up to 2 billion individuals have evidence of exposure to HBV and an estimated 350 million persons worldwide are chronically infected with HBV [1, 2]. Most of these came from East Asia and sub-Saharan Africa [3, 4]. Approximately 470 million inhabitants of Africa are infected with this virus at some time during their lives and about 10% remain infected. Overall prevalence in Ethiopia varies from 4.7-16.8% for Hepatitis B surface antigen (HBsAg) and 70-76.38% for at least one marker positive [5–10].

Viruses are involved in the pathogenesis of type I Diabetes Mellitus (DM) in at least two distinct ways: (a) by directly destroying insulin-producing pancreatic β-cells by cytolytic infection, and (b) by triggering or somehow contributing to β-cell–specific autoimmunity, leading to the development of type I DM [11].

Patients suffering from type I DM incur high risk of infection with hepatotropic viruses because of frequent hospitalization and blood tests [12]. A well-documented, yet under-acknowledged risk associated with blood glucose monitoring is the transmission of blood borne viral pathogens such as HBV [13].

Many chronically infected persons show no outward signs of HBV infection, therefore screening for hepatitis B is necessary to: Identify individuals who have chronic HBV infection so they can receive appropriate medical management; Identify those who are unprotected so they can be vaccinated [14]. Individuals born in areas of high or intermediate prevalence rates for HBV (like Africa and Asia) including immigrants and adopted children should be screened [2, 4].

## Methods

A cross sectional comparative study was conducted at Woldiya Zonal Hospital which is found in north Wollo zone in Amhara region, Ethiopia from November, 2010 to January, 2011. One hundred and eight Diabetic patients with age between 18 and 60 years, who came to the Hospital during the study period, were included in the study. One hundred and eight healthy individuals were included as controls.

Information for socio demographic data, history of exposure for the possible associated factors, type of diabetic and years of follow up (only for diabetic patients) was collected using structured questionnaire. The participant serologic status for HBsAg, SGOT and SGPT levels was done using **VISITECT HBsAg** (UK,Omega) rapid test kit and humastar 80 chemistry analyzer respectively.

Data entered and analyzed using SPSS version16. Variables descriptively expressed as mean ± SD or number and percent. Comparisons between groups made using Student's t test for continuous variables and Chi-square or fisher's exact test for categorical data. A multivariate logistic regression model used to determine the independent effect of various factors that were potentially associated with the risk of hepatitis in both groups. All tests were two-sided with α-level of 0.05 and 80% power.

Ethical clearance was obtained from the Institutional Research and Ethics Committee of College of Health science, Addis Ababa University.

## Results

A total of 216 participants were included in this study, of whom 108(50%) participants were diabetes patients and the rest 108(50%) were non diabetes healthy controls. Sex, weight, level of education, smoking, alcohol consumption and most of clinical characteristics were comparable between diabetes and non diabetes control (Table 1). In the study, 96 (44.4%) were female and 120 (55.6%) were male. Mean age and weight of participants were 33.4 years and 54.3 kg respectively. Of the total 216 study participants, 90(41.7%) were married, 80(37%) were single, 36(16.7%) were divorced and 10(4.6%) were widowed. Majority of participants 80(37%) were illiterate and 91(42.1%) were farmers. With regard to diabetes participants, most of them, 75 (69.4%) were type I diabetes and 33 (30.6%) were type II diabetes. Among the 216 participants, 21(9.7%) had abnormal alanine aminotransferase (ALT) (>40IU/l) and 43(19.9%) had abnormal as aspartate aminotransferase (AST) (>40IU/l) (Table 1). Of the total 216 study participants, 8 (3.7%) subjects were positive for HBsAg, four from each of diabetes (3.7%) and non diabetes (3.7%) indicating that there was no significant difference between the prevalence of HBsAg in diabetes patients and in non diabetes controls (Odds Ratio (OR) = 1.00; 95% CI: 0.244-4.1; p = 1.00). Among positive participants, four were females and the rest four were males showing no difference between female and male participants.

**Table 1:** Comparison of sociodemographic, rate of HBsAg positivity and LFT values between DM patients and control subjects, Woldiya, 2011

	Diabetic patients	Control subjects	p-value
**Age (mean)**	37.6±13	29.2±10.4	0.001
**Weight (mean)**	54.6±4.8	54.1±5	0.824
**Sex (n)**			
*Male*	57 (52.8%)	63 (58.3%)	0.494
*female*	51 (47.2%)	45 (41.7%)	
ALT (Iu/l)	25.2±1	20.5±11.7	0.001
AST (Iu/l)	30.4±13	27.8±15.7	0.001
**HBsAg(n)**			
*Positive*	4 (3.7%)	4 (3.7%)	1.000
*negative*	104 (96.3%)	104 (96.3%)	
**Active smoking (n)**			
*Yes*	1 (1%)	3 (2.8%)	0.614
*no*	107 (99%)	105 (97.2%)	
**Alcohol consumption (n)**			
*Yes*	9 (9.3%)	32 (29.6%)	0.00^*^
*no*	99 (91.7%)	76 (70.4%)	
**Total**	108 (100%)	108 (100%)	

In this study we tried to observe the association of the presence of HBsAg with sociodemographic variables; and clinical characteristics of the study participant using multivariate logistics regression method. Except history of liver disease and invasive procedures, no clinical and sociodemographic characteristics were associated with HBV infection in our study (Table 2 and 3).

**Table 2:** Sociodemographic variables and status of diabetes types related to the risk of HBV, Woldiya, 2011

Sociodemographic characteristics	HBV positive, N (%)	HBV negative, N (%)	AOR	P-value
**Age**				
*18-45*	6 (75)	170 (81.7)	1	0.507
*46-60*	2 (25)	38 (18.3)	1.95 (0.27-13.9)	
**Sex:**				
*Female*	4 (50)	92 (44.3)	1	0.743
*Male*	4 (50)	116 (55.7)	1.38(0.2-9.63)	
**Marital status**				
*Marriage*	4 (50)	86 (43.4)	1	
*Single*	2 (25)	78 (37.5)	0.49(0.003-3.9)	0.501
*Divorced*	2 (25)	34 (16.3)	1.64(0.22-12.4)	0.60
*widowed*	0 (0)	10 (4.8)	NA	0.999
**Educational status**				
*Illiterate*	4 (50)	76 (36.5)	1	
*Primary school*	1 (12.5)	65 (31.2)	0.22(0.02-2.79)	0.245
*Secondary school*	1 (12.5)	19 (9.1)	0.84(0.05-13.7)	0.903
*Diploma and above*	2 (25)	48 (23.1)	0.395(0.03-6.2)	0.509
**Occupation**				
*Has no job*	1 (12.5)	19 (9.1)	1	
*Government employee*	2 (25)	18 (8.7)	3.7(0.168-80.5)	0.409
*Farmer*	3 (37.5)	88 (42.3)	0.34(0.03-4.5)	0.408
*Private employee*	2 (25)	83 (40)	0.39(0.03-6.2)	0.504

**Table 3:** Clinical characteristics and LFT value related to the risk of HBV between HBsAg positive and negative subjects, Woldiya, 2011

Clinical Characteristics	HBsAg positive, N (%)	HBsAg negative, N (%)	AOR	P-value
**History of jaundice(chronic liver disease)**				
*no*	7 (87.5)	206 (99.04)	1	0.022
*Yes*	1 (12.5)	2 (.96)	448(2.45-8.22)	
**History of hospital admission**				
*no*	6 (75)	137 (65.9)	1	0.069
*Yes*	2 (25)	71 (34.1)	0.021(0-1.35)	
**History of surgical operation**				
*no*	6 (75)	186 (89.4)	1	0.599
*Yes*	2 (25)	22 (10.6)	0.42(0.02-10.7)	
**History of blood transfusion**				
*no*	7 (87.5)	201 (96.6)	1	0.099
*Yes*	1 (12.5)	7 (3.4)	128(0.4-4.16)	
**History of hemodialysis (CKD)**				
*no*	7 (87.5)	190 (91.3)	1	0.583
*Yes*	1 (12.5)	18 (8.7)	2.8(0.07-113.9)	
**Receiving corticosteroids or immunosuppressive drugs**				
*no*	7 (87.5)	194 (93.3)	1	0.478
*yes*	1 (12.5)	14 (6.7)	0.26(0.006-10.5)	
**History of household contacts**				
*no*	8 (100)	205 (98.6)	0.999	
*Yes*	0 (0)	3 (1.4)	NA	
**History of multiple sexual partner**				
*no*	6 (75)	141 (67.8)	1	0.799
*Yes*	2 (25)	67 (32.2)	1.32(0.16-10.9)	
Invasive procedures*				
*No*	3(37.5)	145(69.7)	1	0.048
*Yes*	5(62.5)	63(30.3)	9.4(1.02-86.8)	
**GPT**				
*<24 Im/l*	1 (12.5)	125 (60)	1	
*24-40 Im/l*	5 (62.5)	64 (30.8)	9.6(0.23-392)	0.233
*>40 Im/l*	2 (25)	19 (9.2)	2.73(0.04-185)	0.641
**GOT**				
*<24 Im/l*	1 (12.5)	87(41.8)	1	
*24-40 Im/l*	1 (12.5)	84(40.4)	0.402(0.007-23)	0.661
*>40 Im/l*	6 (75)	37(17.8)	9.6 (0.18-502)	0.263

* Abortion, tooth extraction, ear piercing, and tattooing

## Discussion

In this study, the prevalence of HBsAg in diabetic patients and non diabetes controls was the same and estimated to be (3.7% versus 3.7%; Odds Ratio (OR) =1.00; 95% CI: 0.244-4.1; p = 1.00) indicating that there was no significant difference in the prevalence rate of HBsAg seropositivity between diabetic patients and the control population. These findings showed that that the vast majority of patients with diabetes have no increased susceptibility to infection by HBV than the general population. This study also showed that the study area to be of intermediate endemicity (2-8%) with HBV and consistent with previous serologic data from most region of Ethiopia [5, 8–10].

We compared diabetes and non diabetes with some sociodemographic variables and LFT values, except age, all the sociodemographic variables (weight, sex, smoking and alcohol consumption) did not show any difference indicating that the two groups are comparable. The difference age was due to the fact that that controls were VCT clients who came to VCT for marital purpose. The LFT result showed difference between diabetes and non diabetes in this study. The prevalence of high ALT levels may reach 20% in diabetes. Elevation of these enzymes is strongly related to obesity, diabetes and dyslipidemia, and their measurement may act as a surrogate marker of non alcoholic fatty liver disease (NAFLD) presence [15, 16].

Association was obtained between HBsAg positivity and history of invasive procedures like tooth extraction, abortion and body piercing. This association is reasonably accepted. In the study area practice of unsafe tooth extraction, abortion and tattooing is widely common and it agrees with Negero et al (2011) study [10].

History of liver disease was significantly associated with HBsAg. The main cellular target of HBV is the hepatocyte, and in humans, these are the only cells convincingly shown to replicate the virus. HBV is responsible for a chronic hepatitis, leading to cirrhosis and liver cancer in many parts of the world [17]. HBV is causally associated with primary hepatocellular carcinoma (PHC) [18].

The serum alanine aminotransferase was normal in both groups. Normal serum alanine aminotransferase (ALT = 34.8 IU/l ± 13.8 SD) levels in patients with HBsAg in our result may indicate the inactive HBsAg carrier state (After spontaneous HBeAg seroconversion, 67% to 80% of carriers have low or undetectable HBV DNA and normal ALT levels with minimal or no necroinflammation on liver biopsy) [2, 19] or may reflect mild chronic hepatitis B or hepatitis B with fluctuating disease activity [20].

Aspartate aminotransferase was higher than the normal value (AST = 49.8 IU/l ± 25.2 SD) but statistically not different from the sero negative group (AOR = 9.6; 95% CI: 0.18-502; P = 0.26), one of the reasons for this situation might be the effect of diabetes. The presence of diabetes remained an independent risk factor for chronic liver diseases and HCC after adjustment for alcohol use or viral hepatitis in the studies that evaluated these factors [21].

History of transfusion and history of alcohol consumption were not associated with HBsAg in our study. All this results were consistent with Shimelis et al, 2007 study. Transfusion with blood or blood products is no longer an important risk factor for acute viral hepatitis [2, 20].

History of multiple sexual partners did not associate with HBsAg positivity in our study. hemodialysis also did not associate with hepatitis seropositivity in contrast to [22] study. As we can see from Table 2 and 3; the confidence interval is so wide indicating that the sample size is small and the point estimate is imprecise. Because of these reason, we could not strongly conclude that the aforementioned clinical characteristics, laboratory and sociodemographic variables did not associated with HBV infection.

Many previous studies, conducted in various nations, including Taiwan (13.5%Vs12.4%) [23], South Africa (4.6%Vs 4.3%) [24], Turkey (3.4%Vs 2.2%) [25], Nigeria (20% Vs17.3%) [26] and Turkey (5.1%Vs 3.8%) [27] reported a higher prevalence rate of hepatitis B in diabetic patients than non diabetic, but no significant difference was found. Our finding which (3.7% Vs 3.7%) was apparently consistent with the above mentioned studies as there was no statistical difference between diabetes and non diabetes. Unlike other studies our result showed equal prevalence between diabetes and non diabetes. This might be due to small sample size that we used as indicated by wide range of confidence interval; (AOR = 1.00; 95% CI: 0.244-4.1) and chance as indicated by p-value (p = 1.00).

Studies done in Ethiopian on different target population, for example a study done by Abebe et al (2003) [5] in Addis Ababa residents, Shimelis et al (2007) [8] at Saint Paul’‘s General Specialized Hospital on VCT clients, by Tessema et al (2010) [9] at University of Gondar on blood bank and by Negero et al (2011) [10] among VCT clients at Shashemene Hospital showed 7%, 5.7%, 4.7% and 5.7% HBsAg prevalence, respectively. When we compared these finding with ours, they showed intermediate endemicity prevalence like the current study but slightly higher. The possible reason for these differences might be due to differences in method, type of lab test kit used; sample size, geographic distribution and socio demographic variables.

Our low prevalence might also be attributed to the failure to identify infected patients because of the serologic window during the incubation period following infection, the presence of some rare variants escaping the serologic assay for HBsAg, particularly when concurrent testing for anti-HBc is not performed [17] and the problem of occult HBV infections [2, 17] in which neither HBsAg or anti-HBc are detected [17]. Since the assay is serologic, there might be false negative, especially for HBV, patient antibody may be bound with viral antigen in immune complexes, thereby preventing antibody detection [18].

Generally this study and other previous studies conducted in Ethiopia showed lower prevalence than WHO report of greater than 8% for HBsAg. This discrepancy might be due to study designs, because in endemic area of Africa and Asia, most infections occurs in infants and children as a result of maternal-neonatal transmission or close childhood contact [2, 4] and these studies done on adults which have lower prevalence than infants, children and special group of populations who are at special risk making the prevalence of these study lower than WHO report.

## Conclusion

Generally our findings suggest that DM has no any association with HBV infection and the prevalence of HBV is similar in both diabetic cases and control groups and therefore diabetic patients would require no special anti HBV prophylaxis than the general population.
